# Soft, stretchable conductive hydrogels for high-performance electronic implants

**DOI:** 10.1126/sciadv.ads4415

**Published:** 2025-03-21

**Authors:** Md Saifur Rahman, Ahnsei Shon, Rose Joseph, Anton Pavlov, Alex Stefanov, Myeong Namkoong, Heng Guo, Dangnghi Bui, Reid Master, Archita Sharma, Jennifer Lee, Melissa Rivas, Ananya Elati, Yava Jones-Hall, Feng Zhao, Hangue Park, Michelle A. Hook, Limei Tian

**Affiliations:** ^1^ Department of Biomedical Engineering, Center for Remote Health Technologies and Systems, Texas A&M University, College Station, TX 77843, USA.; ^2^ Department of Multidisciplinary Engineering, Texas A&M University, College Station, TX 77843, USA.; ^3^ Department of Neuroscience and Experimental Therapeutics, Texas A&M University, Bryan, TX 77807, USA.; ^4^ Department of Veterinary Pathobiology, School of Veterinary Medicine and Biomedical Sciences, Texas A&M University, College Station, TX 77843, USA.; ^5^ Department of Biomedical Engineering, Sungkyunkwan University, Suwon, South Korea.; ^6^ Department of Intelligent Precision Healthcare Convergence, Sungkyunkwan University, Suwon, South Korea.; ^7^ Department of Electrical & Computer Engineering, Texas A&M University, College Station, TX 77843, USA.

## Abstract

Conductive hydrogels are emerging as promising materials for electronic implants owing to their favorable mechanical and electrical properties. Poly(3,4-ethylenedioxythiophene):poly(styrene sulfonate) (PEDOT:PSS) hydrogels are particularly attractive, but their preparation often requires toxic additives. Here, we introduced a nutritive sweetener, d-sorbitol, as a nontoxic additive to create soft and stretchable PEDOT:PSS conductive hydrogels. These hydrogels exhibit mechanical properties comparable with biological tissues, reducing adverse immune responses. The hydrogels can be patterned on elastic substrates using a simple, low-cost micromolding technique to fabricate soft and stretchable implantable devices for electrical stimulation and recording. The hydrogel electrodes show much lower electrochemical impedance and higher charge storage and injection capacity compared to platinum electrodes. In addition, the properties of hydrogels and devices remain stable after long-term storage and exposure to extreme conditions. We demonstrate the use of soft hydrogel-based electronic devices for effective electrical stimulation and high-quality electrical recordings in live animal models.

## Teaser

Soft conductive hydrogels synthesized with a nutritive sweetener enable effective neural stimulation.

## INTRODUCTION

Electronic implants are widely used to diagnose and treat various diseases, such as epilepsy and Parkinson’s disease, and establish brain-machine interfaces that seek to restore lost motor and sensory function (*1*). The electrodes in these implantable devices play a vital role in achieving high-quality electrophysiological recording and effective electrostimulation without adverse effects on biological systems. Existing implantable electrodes mainly rely on conductive materials with high Young’s modulus, such as platinum (Pt) and silicon (*2*). Mechanical disparity across rigid material-tissue interfaces causes increased immune responses and chronic tissue inflammation (*3*). Consequently, the scar tissue grown on the conductive surface decreases electrical signal recording quality (e.g., signal-to-noise ratio) and stimulation efficacy. Developing biocompatible conductive materials that exhibit tissue-matching mechanical properties with Young’s moduli ranging from 1 kPa to 1 MPa and high stretchability, as well as high electrical conductivity and charge injection capacity, remains challenging in high-performance electronic implantable devices.

To tackle this challenge, electrically conductive hydrogels (CHs) have been developed to offer tissue-like mechanical properties and electronic and ionic dual conductivity for electronic implants (*4*, *5*). Various intrinsically conductive polymers, such as polypyrrole, polyaniline, poly(3,4-ethylenedioxythiophene), have been used in hydrogel-based bioelectronics (*5*). Among these polymers, poly(3,4-ethylenedioxythiophene):poly(styrene sulfonate) (PEDOT:PSS) is particularly attractive because of its tunable electrical property, good solution processability, biocompatibility, and optical transparency (*6*). Various additives, including dimethyl sulfoxide (DMSO), ethylene glycol, ionic liquid, and sulfuric acid, have been used to increase the conductivity of PEDOT:PSS by inducing phase separation between the conductive and hydrophobic moiety PEDOT and insulating and hydrophilic PSS (*7*–*10*). For example, the PEDOT:PSS hydrogels obtained with concentrated sulfuric acid provide an electrical conductivity of 8.8 S/cm measured in pH 1 aqueous solution (*11*). The PEDOT:PSS hydrogels prepared with DMSO and ionic liquid show a high electrical conductivity of 40 to 50 S/cm in water (*7*, *8*). These additives, however, can cause a variety of adverse effects on living organisms (*12*, *13*), which require thorough removal. Laser-induced PEDOT:PSS hydrogels were also developed to enable high electrical conductivity and eliminate the use of additives (*14*). However, the resultant hydrogels exhibit Young’s modulus higher than 50 MPa and ~15% fracture strain. The mechanical properties do not match those of biological tissues, including skin, spinal cord, and peripheral nerves, which can experience as much as 30% tensile strain during routine body movements (*15*).

Here, we introduce a soft, electrically CH composed of interconnected networks of PEDOT:PSS nanofibrils induced by d-sorbitol, a nutritive sweetener. The PEDOT:PSS hydrogel exhibits tunable electrical, mechanical, and swelling properties with varying amounts of d-sorbitol. The CH was prepared by adding d-sorbitol to a PEDOT:PSS solution, drying, annealing, and then swelling in deionized (DI) water or phosphate-buffered saline (PBS). We observed an anisotropic structure and swelling behavior of the PEDOT:PSS film following the drying and annealing process. The CH in water, prepared with 3% (w/v) d-sorbitol, exhibits electrical conductivity of ~74 S cm^−1^, a low tensile modulus of 2.6 MPa and compression modulus of 0.7 MPa, and a high stretchability of 40% strain. The CH can be patterned on elastic substrates to yield soft electrodes with a simple, cost-effective micromolding approach. The CH electrode offers a charge injection capacity of 46.9 mC cm^−2^ for a pulse duration of 100 ms, which is 84 times higher than a Pt electrode (0.56 mC cm^−2^). The impedance of the CH electrode shows up to three orders of magnitude lower than that of the Pt electrode in the physiologically relevant frequency range. These electrochemical properties remain stable after long-term storage and exposure to autoclave conditions. We demonstrated that the CH electrode could provide effective electrostimulation in vivo in a rat model and validated the biocompatibility for chronic implantation. These findings are built upon a series of advances. First, the synthesis of PEDOT:PSS hydrogels with d-sorbitol eliminates additive-associated cytotoxicity. Second, the mechanical, electrical, and electrochemical properties of the CHs are optimized for implantable bioelectronic interfaces. Third, our CH electrodes yield a record-high ankle rotation angle of 51° induced by a low stimulation voltage of 0.1 V (table S1) (*7*, *8*, *14*, *16*–*19*).

## RESULTS

### CH design and patterning

**Fig. 1.**
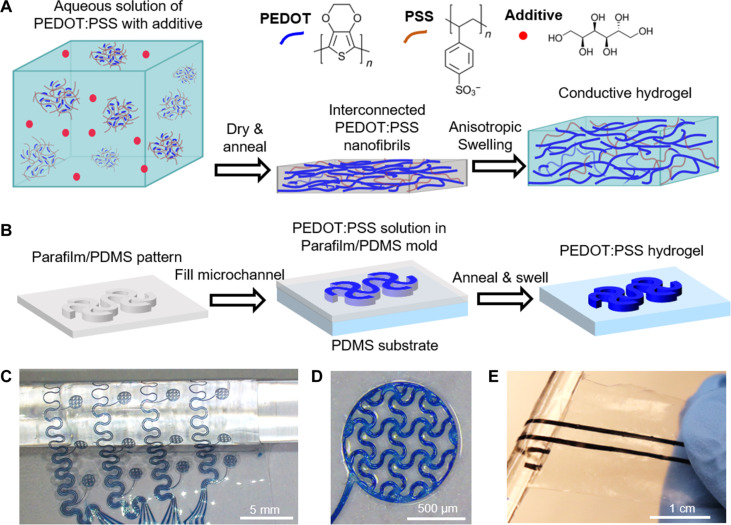
CH design and patterning. (**A**) Schematic illustration of PEDOT:PSS dry film preparation with aqueous PEDOT:PSS solution and d-sorbitol and conversion of the dry film to a stable CH following anisotropic swelling. (**B**) Illustration of patterning CH on a PDMS thin film using a micromolding approach. Optical images of (**C**) a CH mesh electrode array wrapped around a glass rod and (**D**) an individual electrode. (**E**) Optical image of CH lines on a PDMS film patterned with laser-cut Parafilm.

In PEDOT:PSS aqueous colloidal dispersion, hydrophobic PEDOT forms stable complexes with insulating hydrophilic PSS. Pristine aqueous PEDOT:PSS solution without additives results in dry films’ poor mechanical stability in wet environments (*7*, *20*). Our previous work showed that d-sorbitol added to a PEDOT:PSS solution could improve the electrical conductivity, mechanical stretchability, and stability of dry films (*21*). Others have also investigated the electrical and mechanical properties of the dry films treated with d-sorbitol (*22*). d-Sorbitol works as a secondary dopant and plasticizer to induce recrystallization of the PEDOT and rearrangement of PEDOT and PSS chains during high-temperature annealing (*23*, *24*). d-Sorbitol occurs naturally in a variety of berries and fruits and could be a suitable additive to prepare soft conductive materials in electronic implants owing to its excellent biocompatibility. However, the feasibility of using d-sorbitol to obtain PEDOT:PSS hydrogels with low modulus, high electrical conductivity, and long-term stability has not been investigated. Here, we blend d-sorbitol into a PEDOT:PSS solution followed by drying and annealing to facilitate the phase separation between rigid PEDOT-rich domains and soft PSS-rich matrix (Fig. 1A). The recrystallization of PEDOT-rich domains yielded interconnected highly conductive nanofibrils. After immersing the annealed PEDOT:PSS film in water or PBS, the hydrophilic PSS-rich matrix swells over the thickness direction to form a soft, stable hydrogel.

Various types of fabrication approaches, including printing (*7*, *25*, *26*), laser irradiation (*14*), electrochemical patterning (*27*), and photolithography (*8*, *28*), have been used to pattern conductive polymer-based hydrogels as electrodes and interconnects in bioelectronic devices. In this work, we patterned the CH using a facile, low-cost micromolding approach (Fig. 1B). The micromolds can be fabricated in two methods. One method relies on a soft lithography approach to create a micropattern on an ultrathin polydimethylsiloxane (PDMS) film. An SU-8 master was first fabricated via photolithography, and the PDMS precursor was spin coated on the master, followed by curing to form an ultrathin PDMS film. The PDMS film was removed from the master and then modified with polyvinyl alcohol (PVA) to enhance the adhesion between the PEDOT:PSS hydrogel and PDMS film. This method enables the patterning of PEDOT:PSS films with a wire width of 25 μm, in which the feature resolution is defined by photolithography. Although the SU-8 masters are reusable, they still need to be fabricated in the clean room. Alternatively, a flexible, thermoplastic film (Parafilm, ~130 μm in thickness) can be patterned using a laser cutter and used as a mask to create a temporary microchannel. The patterned Parafilm can be seamlessly laminated on the PVA-coated PDMS surface and then removed after drying the PEDOT:PSS solution. This approach can prototype different patterns at a low cost and yield a narrow microchannel width of ~250 μm. In both approaches, a desired pattern of PEDOT:PSS CH can be obtained by filling the microchannels with the d-sorbitol–blended PEDOT:PSS solution, followed by drying at 60°C for 2 hours, annealing at 130°C for 2 hours, and immersion in water or PBS. Figure 1C shows a representative optical image of a PEDOT:PSS CH electrode array on a 150-μm-thick PDMS film prepared with soft lithography and laminated on a glass rod. The electrodes are 1 mm in diameter and composed of serpentine filaments with a wire width of 50 μm (Fig. 1D). Figure 1E shows an optical image of PEDOT:PSS CH lines on a 150-μm-thick PDMS film patterned with Parafilm. The linewidth is 500 μm, and the center-to-center distance is 3 mm.

### Morphology and swelling of the annealed film

**Fig. 2.**
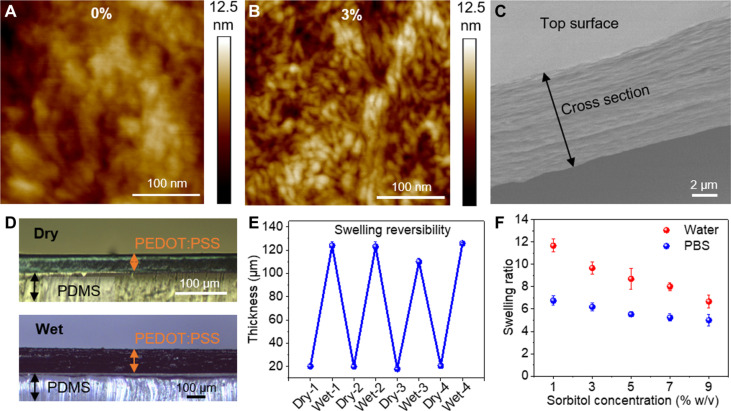
PEDOT:PSS dry film and swelling characterization. (**A**) AFM topography images of a pristine PEDOT:PSS dry film and (**B**) the film obtained with 3% (w/v) d-sorbitol. (**C**) Cross-sectional SEM image of the PEDOT:PSS dry film [3% (w/v) d-sorbitol]. (**D**) Cross-sectional optical images of a PEDOT:PSS dry film and CH on a PDMS substrate. (**E**) Reversible anisotropic swelling of CH (*n* = 3). (**F**) CH swelling ratio in water and PBS with varying d-sorbitol concentrations (*n* = 3). All error bars denote the SD.

We first investigated the effect of varying d-sorbitol concentrations on the morphology of the annealed films using atomic force microscopy (AFM). We prepared the PEDOT:PSS films with d-sorbitol of varying concentrations from 1 to 9% (w/v) following the same process described above. After drying and annealing, the films were immersed in water to remove free d-sorbitol and dried before AFM analysis. The pristine PEDOT:PSS film showed a smooth surface without any sign of phase separation, as shown in the AFM topography image (Fig. 2A and fig. S1A). In comparison, the PEDOT:PSS film with 1% (w/v) d-sorbitol showed fibrous morphology (fig. S1B). This indicates that a small amount of d-sorbitol induced the phase separation between PEDOT-rich and PSS-rich domains during the annealing process and resulted in the formation of PEDOT-rich nanoparticles and nanofibrils. The nanofibril density increased with an increased d-sorbitol concentration of 3% (w/v) (Fig. 2B). The interconnected nanofibrils are expected to substantially increase the electrical conductivity of the PEDOT:PSS film. Further increased d-sorbitol concentration of 9% (w/v) did not show a substantial difference in the morphology and nanofibril density compared to 3% (w/v) d-sorbitol film (fig. S1, C and D). We further characterized the structure of the dry film with 3% (w/v) d-sorbitol using scanning electron microscopy (SEM). The film cross-sectional SEM image revealed in-plane layers stacked over the thickness direction, following the preferential in-plane distribution of PEDOT-rich nanofibrils during the drying and annealing process (Fig. 2C). The anisotropic structure likely results from the anisotropic drying of the PEDOT:PSS aqueous solution. A similar anisotropic structure was also observed in CHs synthesized with ionic liquid (*8*).

Following the anisotropic structure, we observed the anisotropic swelling of the dry film in water and the physiological environment (1X PBS). Figure 2D shows the optical images of the dry film prepared with 3% (w/v) d-sorbitol before and after immersing in PBS. The lateral dimension of the films remained the same whereas the film thickness increased, indicating swelling only occurs in the out-of-plane direction (Fig. 2D and fig. S2). We collected the top-view optical images of a freestanding film to eliminate the substrate effect. The swelling occurs rapidly and reaches equilibrium in 15 s (Fig. 2D). The thickness of dry film with 3% (w/v) d-sorbitol increases by six times from the initial ~20 to ~120 μm after immersing in PBS. The swelling can be restored after repeated drying, indicating the mechanical stability of physically cross-linked PEDOT-rich nanofibrils and PSS-rich matrix (Fig. 2E). We defined the swelling ratio as the ratio of the thickness of the swollen hydrogel to its initial dry film thickness. The swelling ratio is sensitive to the ionic strength of the aqueous environment. The swelling ratio is higher in water than in PBS, likely due to the charge screening effect and reduced charge interactions in PBS (Fig. 2F). The concentration of d-sorbitol in a PEDOT:PSS solution also affects the swelling ratio. The swelling ratio decreases from 6.7 to 5.0 in PBS and from 11.7 to 6.7 in water with increased d-sorbitol concentration from 1 to 9% (w/v) because of the increased density and interconnectivity of PEDOT-rich nanofibrils. The anisotropic swelling behavior and swelling ratio in PBS are similar to the PEDOT:PSS hydrogels prepared with DMSO and ionic liquid reported previously (*7*, *8*). The PEDOT:PSS hydrogels prepared with 3% (w/v) d-sorbitol showed 84 wt% water content. The hydrogels remained mechanically stable after being kept in PBS and water for more than 3 months (fig. S2).

### CH mechanical and electrical properties

**Fig. 3.**
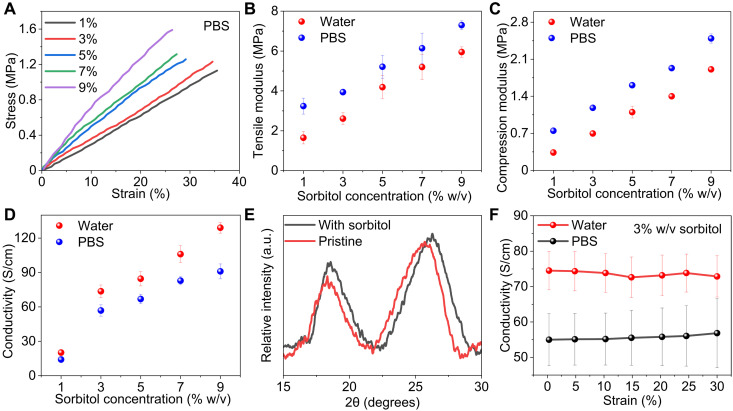
Mechanical and electrical characterizations. (**A**) Tensile stress-strain curves of freestanding CH in PBS with varying d-sorbitol concentrations. (**B**) CH tensile modulus and (**C**) compressive modulus versus d-sorbitol concentration in water and PBS (*n* = 3). (**D**) CH electrical conductivity in water and PBS with varying d-sorbitol concentrations (*n* = 3). (**E**) XRD patterns of pristine PEDOT:PSS film and the film prepared with 3% (w/v) d-sorbitol. a.u., arbitrary units. (**F**) CH electrical conductivity in water and PBS at different strains (*n* = 3). All error bars denote the SD.

We characterized the CH mechanical properties by the tensile and compression measurements of freestanding hydrogel films in PBS and water. Figure 3A shows the tensile stress-strain curves of hydrogel films prepared with varying d-sorbitol concentrations in PBS. The tensile modulus linearly increases from 3.2 to 7.3 MPa, whereas the ultimate tensile strain decreases from 35 to 26% with increasing d-sorbitol from 1 to 9% (w/v) (Fig. 3, A and B). In comparison, the compression modulus increases from 0.7 to 2.5 MPa (Fig. 3C). The increased moduli originate from the increased density and physical cross-linking of the PEDOT-rich nanofibrils induced by the increased d-sorbitol. The tensile moduli are three to five times higher than compression moduli, following the anisotropic structure of the CH. The mechanical measurements of the CH in water show that tensile modulus linearly increases from 1.7 to 6.0 MPa (Fig. 3B and fig. S3) and compression modulus linearly increases from 0.3 to 1.9 MPa with increasing D-sorbitol (Fig. 3C). The lower moduli of the CH in water compared to PBS results from the higher swelling ratio in water shown in Fig. 2F and the decreased volumetric nanofibril density. The moduli of the PEDOT:PSS hydrogels are comparable to those of soft biological tissues, such as skin (in the range of 0.5 to 3.7 MPa) and peripheral nerves (in the range of 5 to 14 MPa) (*15*, *29*–*31*). The moduli are more than four orders of magnitude lower than those of conventional conductive materials, including platinum, silicon, tungsten, and stainless steel, used in bioelectronic devices (*5*, *32*).

We systematically studied the electrical conductivity of the CH films with different d-sorbitol concentrations (Fig. 3D). The dry and annealed PEDOT:PSS films were immersed in PBS and water overnight before measuring the conductivity using a four-probe method. The data show that the hydrogel electrical conductivity increases from 14 to 91 S/cm in PBS and from 20 to 129 S/cm in water with increasing d-sorbitol concentrations from 1 to 9% (w/v). The hydrogels exhibit overall higher electrical conductivity in water than in PBS. The PEDOT:PSS hydrogels are acidic in water because of the sulfonic acid groups in PSS. The neutralization of acidic PEDOT:PSS with PBS or an alkaline environment decreases the electrical conductivity due to the shift from bipolarons of PEDOT to polarons and disturbance of the π-π stacking of PEDOT crystalline structures (*7*, *33*). For bioelectronic applications, conductive materials with high electrical conductivity and low stiffness are desired. Considering the trade-off between these two properties with increasing d-sorbitol concentrations, we chose the PEDOT:PSS hydrogels prepared with 3% (w/v) d-sorbitol for further characterization and device preparation for peripheral nerve stimulations. The tensile and compression moduli of the PEDOT:PSS hydrogels prepared with 3% (w/v) d-sorbitol are 3.9 and 1.2 MPa, lower than that of peripheral nerves. The electrical conductivity is 74 S/cm in water, higher than the previously reported conductivity of the PEDOT:PSS hydrogels prepared with DMSO (40 S/cm) (*7*) and with ionic liquid (47 S/cm) (*8*), among the highest values for CH (*5*). The CH electrical conductivity showed negligible change after keeping the CH in water for 1 month, confirming the hydrogel stability (fig. S4). We investigated the mechanism of conductivity enhancement in PEDOT:PSS films using x-ray diffraction (XRD) and Raman spectroscopy. The XRD peak around 26° corresponds to the PEDOT thiophene ring π-π stacking (*34*), which shifts from 25.6° for pristine PEDOT:PSS to 26.3° for the film prepared with d-sorbitol (Fig. 3E). The π-π stacking distance reduces from 3.5 to 3.4 Å with d-sorbitol treatment, which can markedly increase the electrical conductivity by improving the interchain charge transfer. The Raman spectra were also collected from pristine PEDOT:PSS and films prepared with d-sorbitol (fig. S5). The Raman bands between 1400 and 1500 cm^−1^ are attributed to the stretching vibration of C_α_═C_β_ on the five-member thiophene ring of the PEDOT chains (*35*, *36*). The C_α_═C_β_ band shifted from 1424 to 1417 cm^−1^ due to the d-sorbitol treatment, which suggests the change in the resonant structure of PEDOT chains from a benzoid to a quinoid structure. This transformation of the resonant structure is associated with the conformation change of the PEDOT chains from the coil to an expanded coil or linear conformation (*36*). The linear conformation enhances charge carrier mobility between the PEDOT chains, resulting in high electrical conductivity. We further characterized the effect of the strain on the CH electrical conductivity under 0 to 30% tensile strain. The electrical conductivity remains stable after 500 stretch and release cycles at 30% strain, which confirms that the CH can match the stretchability of neural tissues to facilitate a seamless electrode-tissue interface (Fig. 3F and fig. S6).

### Electrochemical properties of the CH electrode

**Fig. 4.**
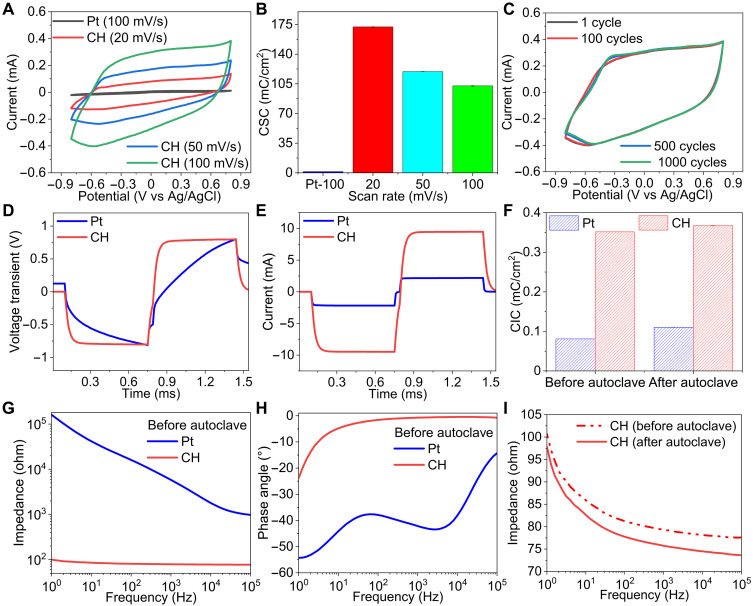
Electrochemical characterization of 3% (w/v) d-sorbitol CH in PBS. (**A**) CV curves of CH and Pt electrodes recorded with different scan rates. (**B**) CSC of CH and Pt electrodes with different scan rates (*n* = 3). (**C**) CH curves with a scan rate of 100 mV/s and increased cycles. (**D**) Voltage transient curves of CH and Pt electrodes and (**E**) corresponding current pulses. (**F**) CIC of CH and Pt electrodes before and after autoclave (*n* = 3). (**G**) Impedance magnitude and (**H**) phase angle plots of CH and Pt electrodes with varying frequency. (**I**) Impedance magnitude of CH electrode before and after autoclave. All error bars denote the SD.

High charge storage capacity (CSC), charge injection capacity (CIC), and long-term stability of electrode materials under physiological conditions are critical for effective electrical stimulation and neuromodulation. We prepared CH electrodes to evaluate these parameters in comparison with standard Pt electrodes of the same electrode dimension (7 mm in length and 0.5 mm in width). The thicknesses of dehydrated CH and Pt electrodes are ~20 and 25 μm, respectively. We encapsulated the interconnect region of both CH and Pt electrodes with a 50-μm-thick layer of PDMS to define the electrode dimension. The electrodes were immersed in PBS for electrochemical characterization, and the experimental setup is shown in fig. S7A. Figure 4A shows the cyclic voltammetry (CV) curves collected from CH electrodes with different sweep rates of 20, 50, and 100 mV s^−1^ and a Pt electrode with a sweep rate of 100 mV s^−1^. The CSC of the Pt electrode is 1.5 mC cm^−2^, which is 69 times lower than that of CH electrodes (103.7 mC cm^−2^) at a sweeping rate of 100 mV/s (Fig. 4B). At a low sweep rate of 20 mV/s, the hydrogel electrodes show an increased CSC of 172.2 mC cm^−2^. This value is higher than those of other PEDOT-based hydrogels, including a PEDOT-PVA-heparin CH (80 mC cm^−2^) (*37*), a PEDOT:PSS hydrogel treated with DMSO (60 mC cm^−2^) (*7*) and a PEDOT:PSS hydrogel treated with an ionic liquid (164 mC cm^−2^) (*8*). In addition, our CH electrodes showed consistent current profiles during 1000 CV cycles and ~3% change in CSC (Fig. 4C and fig. S7B). The CH electrodes sustained high CSC with 3% change after 30 days of incubation in PBS, confirming their electrochemical stability (fig. S7, C and D). It is worth noting that the electrochemical properties of hydrogel electrodes are thickness dependent. The CSC of CH electrodes increased from 64 to 312.8 mC cm^−2^ at a sweep rate of 100 mV/s with increased thickness from 60 to 240 μm (fig. S8, A and B).

We measured voltage transients to determine the CIC by injecting biphasic, cathodal first, and symmetric current. The current limit was set for safe reversible charge injection when the maximum cathodal and anodal electrochemical potentials reached the boundaries of the water hydrolysis window. The maximum cathodal and anodal electrochemical potentials were determined as the potentials at 10 μs after the cathodal and anodal pulses end, following the previously reported protocol (*38*). The water hydrolysis windows span from −0.6 to 0.8 V for Pt and from −0.9 to 0.6 V for PEDOT:PSS (*39*). Figure 4D shows the voltage transient curves of hydrogel and Pt electrodes, following the current pulses with a pulse duration of 0.65 ms, as shown in Fig. 4E. The hydrogel charge injection was limited by polarization to 0.6 V in the anodal phase, whereas Pt was limited by polarization to −0.6 V in the cathodal phase. The CIC of the CH electrode reached 0.35 mC cm^−2^, more than four times higher than that of the Pt electrode (0.08 mC cm^−2^). For a long pulse duration of 100 ms, the CIC of the CH electrode increased to 46.91 mC cm^−2^, 84 times higher than that of the Pt electrode (fig. S9, A and B). The CIC of our CH electrodes is ~6 times higher than that of the PEDOT:PSS CH electrodes treated with DMSO (8.3 mC cm^−2^) (*7*) for the 100-ms pulse duration. In addition, we measured the CIC of CH electrodes with varying thicknesses (fig. S8, D to I). For the short pulse duration of 0.65 ms, the CIC of the ~240-μm-thick CH electrode is 0.46 mC cm^−2^, ~1.3 and 2.4 times higher than that of electrodes with a thickness of ~120 and ~60 μm, respectively. For the longer pulse duration of 100 ms, the CIC of the ~240-μm-thick CH electrode is 63 mC cm^−2^, ~1.3 and 3.2 times higher than that of the ~120- and ~60-μm-thick electrodes, respectively. To evaluate the effects of autoclaving on the electrochemical properties of electrodes, we measured the CIC before and after autoclaving the electrodes and observed no substantial changes (Fig. 4F and fig. S9C). The CIC increase for the CH electrode is 4%, confirming the electrochemical stability of the CH electrode after exposure to extreme conditions (i.e., 121°C saturated steam for 30 min) and the robustness of the PDMS encapsulation.

A low electrode impedance is important for obtaining a high signal-to-noise ratio in biopotential recordings (*39*). We used electrochemical impedance spectroscopy (EIS) to characterize the CH electrode impedance in PBS and compare it with the Pt electrode (Fig. 4G). The impedance of the CH electrode shows up to three orders of magnitude lower than that of the Pt electrode in the frequency range from 1 Hz to 100 kHz, covering typical action potential firing rates. The CH electrode impedance magnitude at 1 kHz, commonly used to evaluate the electrical performance of recording electrodes (*39*), is 79.3 ohms, which is 74 times lower than the Pt electrode impedance. The phase angles of the CH electrode are much lower than those of the Pt electrode (Fig. 4H). The phase angle of the CH electrode is closer to 0° with increasing frequency, which suggests a resistance-dominated impedance. The impedance of CH electrodes can be further reduced by increasing the hydrogel thickness (fig. S8C). The much lower impedance of CH electrodes than Pt electrodes results from the combined electronic and ionic conductivity and high electrochemical surface area of the porous hydrogel electrode. We assessed the impedance magnitude and phase angle after autoclaving both CH and Pt electrodes and found that the results were similar to those recorded before autoclaving (fig. S9, D and E). The impedance magnitude of the CH electrode slightly decreased by 5% at 1 kHz, and the phase angle showed negligible change after autoclaving (Fig. 4I and fig. S9F). In addition, the impedance showed negligible change after soaking in water for 1 month (fig. S10). These results confirm the low impedance, high storage and injection capacity, and electrochemical stability of the CH electrode. These electrochemical properties can be further enhanced by increasing the d-sorbitol concentration in CH synthesis (figs. S11 and S12). The spatially defined, encapsulated electrode is important for evoking fascicle-specific stimulation (*40*). We confirmed that encapsulated CH electrodes on PDMS showed comparable electrochemical properties as the CH electrode with a gold backing and the freestanding CH electrode (figs. S13 and S14). The CH electrode without metal backing could eliminate metallic irritation-induced granulomatous inflammation and a phagocytic response from neutrophils and macrophages in the fibrous tissue surrounding the electrode for neural stimulation (*41*).

### CH biocompatibility

**Fig. 5.**
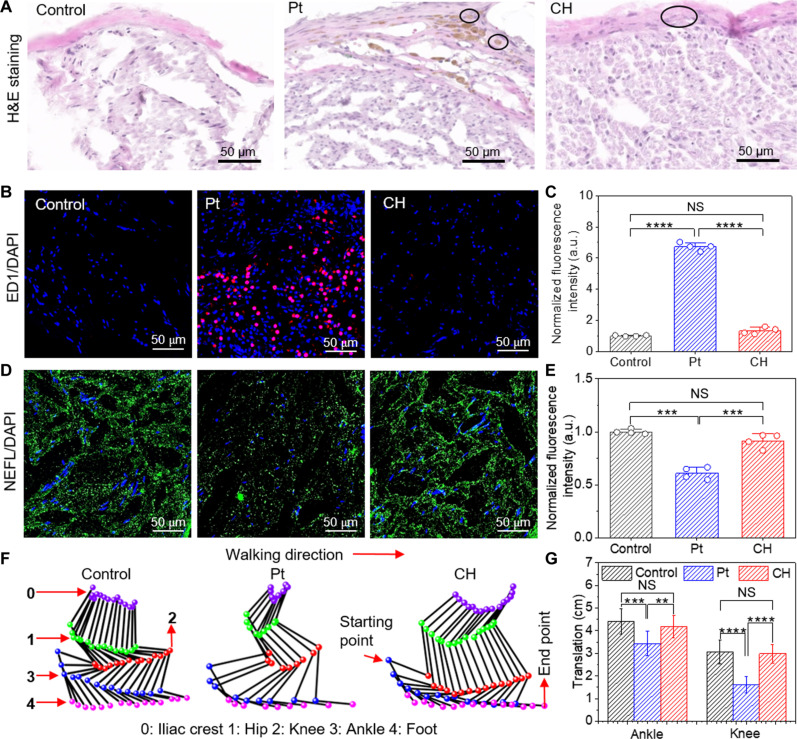
Biocompatibility and rat kinematic analysis. (**A**) H&E-stained cross-sectional slices of the sciatic nerve in contact with CH and Pt electrodes compared with the control slice after 9 weeks of implantation. (**B**) Immunofluorescence images of cross-sectional slices of the sciatic nerve with ED1 labeled in red and DAPI labeled in blue. (**C**) Normalized mean fluorescence intensity of labeled ED1 for CH and Pt electrodes using the control. The *P* values for comparison of the ED1 intensities are as follows: Pt electrode versus control, *P* = 1.553 × 10^−5^; CH electrode versus control, *P* = 0.055, and CH versus Pt electrode, *P* = 6.799 × 10^−8^ (*n* = 4). (**D**) Immunofluorescence images of cross-sectional slices of the sciatic nerve with NF labeled in green and DAPI labeled in blue. (**E**) Normalized mean fluorescence intensity of labeled NF for CH and Pt electrodes using the control. The *P* values for comparison of the NF intensities are as follows: Pt electrode versus control, *P* = 2.327 × 10^−4^; CH electrode versus control, *P* = 0.077, and CH versus Pt electrode, *P* = 4.670 × 10^−4^ (*n* = 4). (**F**) Representative stick diagrams of hindlimb movements for kinematic analysis. (**G**) Ankle and knee translations of the rats with CH and Pt electrodes compared with the control. The *P* values for comparison of the ankle translation are as follows: Pt electrode versus control, *P* = 0.001, CH electrode versus control, *P* = 0.368, and CH versus Pt electrode, *P* = 0.006. The *P* values for comparison of the knee translation are as follows: Pt electrode versus control, *P* = 1.656 × 10^−6^, CH electrode versus control, *P* = 0.842, and CH versus Pt electrode, *P* = 2.218 × 10^−7^ (*n* = 10). All error bars denote the SD, and NS represents not significant. **P ≤ 0.01; ***P ≤ 0.001; ****P ≤ 0.0001.

We first performed cytotoxicity testing to evaluate the CH biocompatibility using in vitro cell cultures of human dermal fibroblasts (hDFs). The viability and morphology of fibroblasts remained consistent after 72 hours of culture with a hydrogel-conditioned medium (fig. S15, A and B). We conducted a 3-(4,5-dimethylthiazol-2-yl)-2,5-diphenyltetrazolium bromide (MTT) assay to quantify cell viability in the presence of a low concentration of 0.5% (w/v) additives (fig. S15C). The evaluated additives include d-sorbitol, as well as three previously reported additives: ionic liquid, glycerol, and DMSO. d-sorbitol had a negligible effect on cell viability, whereas the other additives substantially reduced cell viability, confirming the cytocompatibility of d-sorbitol. For in vivo testing, we wrapped the CH electrode around the sciatic nerve of freely moving rats and evaluated the biocompatibility after 9 weeks of implantation. For comparison, the Pt electrode with the same dimension was also implanted. Figure 5A shows hematoxylin and eosin (H&E)–stained cross sections of the sciatic nerve for histological analysis. With the Pt electrode surrounding the nerve, macrophagic and lymphoplasmacytic inflammation expands in the connective tissue sheath, and the macrophages have brown, granular material in their cytoplasm. In comparison, the sciatic nerve interfaced with the CH electrode showed mild nonsuppurative inflammation in the sheath. We also characterized the degree of immune response by examining the expression of an inflammatory biomarker, ED1. Figure 5B shows the confocal fluorescence images of the tissue slides labeled by anti-ED1 antibodies. For the Pt electrode, the fluorescence intensity is much higher than that in the control slice (intact rat), indicating severe inflammation (Fig. 5C). In contrast, no significant change in ED1 expression was observed for the CH electrode. To examine the nerve damage, we measured the level of neurofilament (NF) in the tissue slides labeled by anti-NF antibodies (Fig. 5D). For the Pt electrode, NFs substantially reduced in the sciatic nerve compared to the control whereas the change induced by the CH electrode was not significant (Fig. 5E). In addition, we performed hindlimb kinematic analysis to examine the gait behavior of the rats after 9 weeks of implantation. We placed five reflective markers on the iliac crest, hip, knee, ankle, and foot of each rat and captured their spatial changes while the rats walked on a treadmill (fig. S16). Figure 5F shows representative hindlimb diagrams of the rats during one stride. The rats implanted with the CH electrode showed comparable ankle and knee translations to the control rats. In contrast, the rats with the Pt electrode showed constrained ankle and knee translations, symptoms commonly associated with severe sciatic nerve dysfunction, such as nerve injuries or inflammatory responses (*42*). These findings underscore the impact of the mechanical mismatch between the rigid Pt electrode and soft nerve tissue, which can impair nerve functionality. In comparison, the CH electrode, with its tissue-matching modulus, induced substantially lower inflammation and preserved normal locomotor function in the rats.

### In vivo neural stimulation and compound muscle action potential recording

**Fig. 6.**
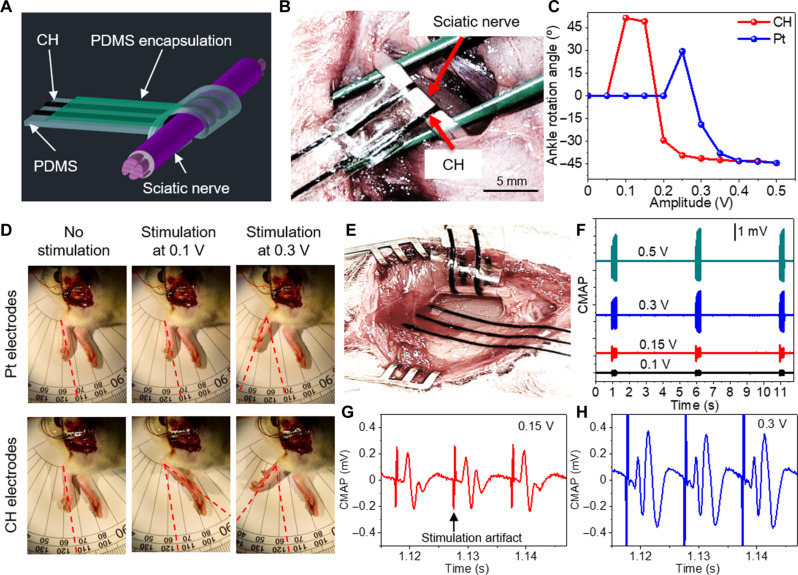
In vivo neural stimulation and simultaneous CMAP recording. (**A**) Schematic of the encapsulated CH electrode wrapped around a sciatic nerve for stimulation application. (**B**) Optical image of the CH electrode wrapped around the sciatic nerve bundle in a live rat. (**C**) Ankle rotation angles induced by the stimulation at 100 Hz with varying voltage amplitudes for CH and Pt electrodes with the same exposed area (*n* = 3). (**D**) Optical Images showing leg movement under different stimulation voltages. (**E**) Optical image of CH electrodes wrapped around the sciatic nerve and laminated on the nearby muscle surface for simultaneous stimulation and CMAP recording. (**F**) CMAP following three pulse trains with varying stimulation voltages. CMAP waveforms with (**G**) 0.15-V stimulation and (**H**) 0.3-V stimulation. All error bars denote the SD.

We prepared the CH and Pt electrodes of the same configuration and compared their performance for neural stimulation. The stimulation devices were prepared on a 100-μm-thick PDMS substrate with a conductive linewidth of 500 μm, a length of 2 cm, and a center-to-center distance of 3 mm (fig. S17, A and B). In the CH device, we used the CH for both electrodes and interconnects without a metal backing. The interconnect regions were encapsulated with a 50-μm-thick PDMS to expose the conductive area of 500 μm by 5 mm to interface with the rat sciatic nerve (Fig. 6A and fig. S17A). The PDMS encapsulation and substrates were treated with oxygen plasma to form a seamless encapsulation of interconnects. The stimulation electrodes were wrapped around the sciatic nerve with a diameter of ~1 mm, which resulted in an effective contact area of 0.8 mm^2^ for each electrode (Fig. 6B). We quantified the ankle rotation angles following the delivery of biphasic symmetric square wave voltage pulses with a pulse duration of 200 μs at 100 Hz to the sciatic nerve (Fig. 6C). For both types of electrodes, we observed dorsiflexion at low stimulation amplitudes and plantar flexion at higher amplitudes. The dorsiflexion was quantified by positive rotation angles, and plantar flexion was quantified by negative rotation angles. This change in ankle rotation direction is likely because the stimulation electrode was primarily in contact with the peroneal fascicle of the sciatic nerve, which innervates the tibialis anterior muscle and produces dorsiflexion at low stimulation amplitudes. With increasing stimulus, the tibial fascicle was also activated, which resulted in a stronger output of gastrocnemius muscle than the tibialis anterior muscle and plantar flexion accordingly. This observation that the increased stimulation intensity changes ankle rotation from dorsiflexion to plantar flexion is consistent with a previous report (*43*). The stimulation with the CH electrode at a low voltage of 0.1 V yielded a high dorsiflexion angle of 51°. The record-high ankle rotation angle results from the higher CSC and CIC of the CH electrodes compared to previously reported hydrogel electrodes (table S1). In comparison, no response was obtained with the Pt electrode at the same stimulation intensity, and a lower dorsiflexion angle of 29° followed the stimulation at 0.25 V (Fig. 6, C and D). Following the stimulation at 0.3 V, the CH electrode resulted in a plantar flexion angle of −41°, higher than that with the Pt electrode (−19°). These results show that the CH electrode can provide higher stimulation efficacy than the Pt electrode. We also used CH electrodes to simultaneously record compound muscle action potential (CMAP) evoked by the nerve stimulation. The recording device comprised recording, reference, and ground electrodes, which was conformably laminated on the muscle surface near the sciatic nerve (Fig. 6E). Figure 6F shows the simultaneously recorded CMAP following three pulse trains with varying stimulation amplitudes from 0.1 to 0.5 V. We observed the increased CMAP with increasing the stimulation amplitudes. The peak-to-peak CMAP increased from 0.4 to 0.7 mV when increasing the stimulation amplitudes from 0.15 to 0.5 V (Fig. 6, G and H). In addition, we compared the CMAP recorded with CH and Pt electrodes (fig. S18). The signal-to-noise ratio of CMAP recordings with CH electrodes is 57 dB, higher than that with Pt electrodes (53 dB). The noise with CH electrodes is 9 μV, lower than Pt electrodes (15 μV). The superior signal quality with CH electrodes mainly results from low electrode impedance. The data demonstrate that CH-based implantable devices can be used for both effective stimulation and high-quality recordings.

## DISCUSSION

The materials and microfabrication method reported here could serve as a useful technology for developing soft electronic devices for neurostimulation and recording. The preparation of the CH eliminates the use of toxic chemicals. The CH provides biological tissue–matching mechanical properties with low modulus and high stretchability. The substantially reduced mechanical disparity at the bioelectronic interface improves mechanical and electrical coupling and reduces the immune response and chronic inflammation of biological systems. The CH can be patterned on an elastomeric substrate with a simple, low-cost micromolding approach to yield soft, stretchable electrodes and interconnects. The high electrical conductivity, charge storage, and injection capacities of the CH electrode enable effective stimulation, which could reduce stimulation-associated adverse effects on the biological tissue. In addition, we show that the CH electrodes can simultaneously record high-quality CMAP. Future opportunities include the integration of recording capability for closed-loop neuromodulation to facilitate the function recovery or substitution for individuals suffering from traumatic or neurological injuries.

## MATERIALS AND METHODS

### Materials

PEDOT:PSS aqueous solution (Clevios PH1000) was purchased from Heraeus Epurio LLC. PDMS (Sylgard184) was obtained from Dow Corning. PVA (molecular weight: ~61,000) was purchased from Sigma-Aldrich. Bovine serum albumin was purchased from Molecular Bioscience. d-Sorbitol and Triton X-100 were purchased from Thermo Scientific Chemicals. Primary antibodies, anti-NF-L rabbit polyclonal antibody (PA5-34650), the LIVE/DEAD Viability/Cytotoxicity Kit (L3224), and 10X PBS were purchased from Invitrogen. Anti-Macrophages/Monocytes Antibody clone ED-1 (MAB1435) was purchased from EMD Millipore. Secondary antibodies, anti-rabbit immunoglobulin G (IgG) H&L (Alexa Fluor 488, ab150077), and goat anti-mouse IgG H&L preadsorbed (Cy5, ab6563) were purchased from Abcam. Vectashield mounting medium with 4′,6-diamidino-2-phenylindole (DAPI) was purchased from Vector Laboratories. Platinum foil, paraformaldehyde (4% in PBS), Dulbecco’s modified Eagle’s medium (DMEM), Dulbecco’s PBS (DPBS), and fetal bovine serum (FBS) were purchased from Thermo Fisher Scientific. An optimal cutting temperature (OCT) compound was purchased from VWR International. Kwik-Sil surgical adhesive was purchased from World Precision Instruments. Aqueous solutions were prepared with DI water (18.2 megohm·cm) produced by the Sartorius Arium Pro Ultrapure water system. All chemicals were used as purchased.

### Fabrication of CHs and electrodes

d-Sorbitol was mixed with a pristine PEDOT:PSS aqueous solution (1.1 to 1.3 wt %) to obtain the PEDOT:PSS solution with varying d-sorbitol concentrations of 1, 3, 5, 7, and 9% (w/v). The mixture was vigorously vortexed for 2 min, sonicated for 20 min, and shaken for 20 min. To prepare freestanding films, the d-sorbitol–blended PEDOT:PSS solution was drop cast on a PDMS substrate, dried at 60°C for 2 hours, and annealed at 130°C. To pattern line electrodes on a PDMS thin film, the PDMS base polymer and curing agent were mixed at a ratio of 10:1, spin coated on a polyimide substrate with 500 rpm for 1 min, and cured at 125°C for 20 min. After curing, the PDMS surface was treated with oxygen plasma and then covered with 1% (w/v) PVA aqueous solution for 10 min, then rinsed with DI water, and dried at 110°C for 10 min. The PVA coating serves as an adhesive between the PEDOT:PSS and the PDMS surface. The Parafilm with open line channels was obtained by a laser cutter (Laser Engraver, Boss LS-1630G) and laminated on the PVA-coated PDMS surface. A gentle pressure was applied to eliminate the air gap between the PVA and Parafilm pattern. The sorbitol-blended PEDOT:PSS solution filled the channels, and the Parafilm pattern was removed once the solution dried. The mesh electrodes were patterned following the protocol previously reported (*21*, *44*). The solution was drop cast onto the PVA-coated PDMS mold, and the excess solution was removed with a soft PDMS blade. The patterned PEDOT:PSS on the PDMS were dried at 60°C for 2 hours and then annealed at 130°C for 2 hours. The annealed freestanding films and patterned electrodes were immersed in DI water or 1X PBS to transform into hydrogels.

### PEDOT:PSS dry film and hydrogel characterization

The topography of dry films prepared with varying d-sorbitol concentrations was collected using an atomic force microscope (Dimension Icon AFM). SEM images of the PEDOT:PSS film were obtained with an ultrahigh-resolution field-emission scanning electron microscope (JEOL JSM-7500F) after lyophilizing the PEDOT:PSS hydrogel using a freeze dryer. Raman spectra of dry films were collected using a DXR Raman spectrometer with a 780-nm wavelength and a 24-mW diode laser as the illumination source. XRD measurement of dry films was conducted using BRUKER D8 x-ray with the x-ray wavelength of 1.54056 Å.

We determined the swelling ratio of the hydrogel films using the following formula. The thickness of the wet films was measured using an optical microscope after the films were immersed in DI water and 1X PBS, respectively. After drying the hydrogel films overnight, the thickness of the dry film was measured

$$\text{Swelling ratio}=\frac{\text{Wet film thickness}}{\text{Dry film thickness}}$$ (1)

The stress-strain curves of the hydrogel films immersed in 1X PBS and DI water were collected using MARK-10. The applied strain rate during the tensile testing was 5 mm/min. We performed compression testing using a nanoindentor (Pavone, Optics11) using a probe with a tip diameter of 9 μm and cantilever stiffness of 3.72 N/m. Optics 11 Data Viewer application was used to analyze the force curve. Compression modulus was quantified using a Hertzian contact fit with a Poisson’s ratio of 0.44. A conventional four-point probe technique with a digital multimeter (NI-USB4065) was used to record the resistance and calculate the conductivity of the hydrogel films after immersion in PBS and DI water overnight.

### Electrochemical characterization of the CH and Pt electrodes

The CV, voltage transient, and EIS measurements were performed with a Gamry Reference 600+ potentiostat in 1X PBS. A three-electrode system was used where Pt mesh was used as a counter electrode, and Ag/AgCl was used as a reference electrode. A freestanding Pt sheet was patterned with a laser cutter (LPKF ProtoLaser U4) to yield electrodes on a PDMS substrate. The thickness of the Pt sheet is 25 μm, close to that of the dry PEDOT:PSS film (~20 μm). A working electrode was an encapsulated CH or Pt film with an exposed geometric surface area (GSA) of 0.035 cm^2^. The CV scanning was conducted with varying scan rates of 20, 50, and 100 mV s^−1^. The CSC was determined by the time integral of the cathodal currents over the potential range from −0.8 to 0.8 V. Voltage transient was measured with two pulse durations of 0.65 and 100 ms. We analyzed the CIC by applying the biphasic symmetric, cathodal first current pulses over the working and counter electrodes. The maximum cathodal and anodal electrochemical potentials were determined as the potentials at 10 μs after the cathodal and anodal pulses end, following the previously reported protocol (*38*). CIC was calculated using the following equation, where *Q*_inj_ = *Q*_inj(c)_ + *Q*_inj(a)_

$$\text{CIC}=\frac{Q_{\text{inj}}}{\text{exposed GSA}}$$ (2)

Impedance was analyzed by conducting the EIS with a frequency range from 1 Hz to 100 kHz and a potential bias of 10 mV.

### CH biocompatibility characterization

The cytotoxicity of hydrogels was assessed using hDFs. Briefly, hDFs at a cell density of 0.1 M cells per well cultured in DMEM and F-12 supplemented with 20% FBS in 24-well tissue culture plates for 24 hours at 37°C in a 5% CO_2_ incubator. CHs of 1 cm^2^ were immersed into 1 ml of fresh hDF cell culture media and left undisturbed for 24 hours to generate the CH-conditioned media. After 24 hours of incubation, the cell media was replaced with CH-conditioned media and incubated for 72 hours. After 72 hours, the media was removed, and cells were washed with DPBS solution. The LIVE/DEAD Viability/Cytotoxicity Kit (L3224; Invitrogen, USA) was applied by adding 4 μM ethidium homodimer-1 and 2 μM calcein AM to the cells and incubated for 15 min at room temperature (*45*). The live (green) or dead (red) cells were observed under a Zeiss fluorescence microscope. In the MTT assay, hDF cells (~5000) were seeded in each well (*n* = 3 for each condition) and exposed to the additive-containing media. After 24 hours, 100 μl of MTT (0.5 mg/ml) in PBS was added and incubated for 4 hours at 37°C. Following incubation, the MTT solution was removed, and 100 μl of DMSO was added to dissolve the resulting MTT formazan crystals. Absorbance was measured at 570 nm using a Cytation 5 microplate reader and normalized to the control group. Statistical analysis was performed using an unpaired, two-sample *t* test.

For long-term implantation, we applied a thin layer of biocompatible silicone adhesive (Kwik-Sil) to secure both CH and platinum electrodes onto the sciatic nerve. The adhesive might still induce mild inflammation, depending on the thickness and application procedure, which should be handled with care. The sciatic nerve tissues (*n* = 4) at the implantation sites of CH and Pt electrodes, as well as the control, were stained by H&E, anti-NF, and anti-ED1 antibodies to examine the nerve damage and immune response. All the rats used for biocompatibility were euthanized 9 weeks after implantation, and the nerve tissues were harvested and fixed with 4% paraformaldehyde for 24 hours at room temperature. The fixed samples were transferred to 30% (w/v) sucrose aqueous solution and kept overnight. The samples were then transferred to an OCT solution for 8 hours and then frozen at −80°C. The frozen samples were sectioned into 15-μm-thick tissue slices using a cryosectioning instrument (Leica CM1860 UV). For histological analysis, slices (*n* = 3) for each condition were stained with H&E. The NF and ED1 proteins in the sciatic nerve tissue were analyzed through immunostaining of the nerve tissue slices. The cryosectioned samples were fixed using absolute ethanol at −20°C for 10 min. The slices were then washed three times with 1X PBS and permeabilized by 0.1% Triton X-100 in PBS for 15 min. The samples were incubated in 3% (w/v) bovine serum albumin and 0.1% Triton X-100 in 1X PBS to minimize nonspecific binding for 45 min. The samples were stained separately with primary antibodies anti-NF (dilution 1:200) and anti-ED1 (dilution 1:200) in a blocking solution overnight at 4°C. Before staining with the secondary antibody, the samples were washed with 1X PBS. The samples were then exposed to secondary antibody anti-rabbit IgG H&L (1:1000, Alexa Fluor 488, ab150077) and goat anti-mouse IgG H&L preadsorbed (1:1000, Cy5, ab6563) for 1 hour. After washing the samples with 1X PBS, nuclei were stained using DAPI. The stained samples were imaged, and the fluorescence intensity of the tissue slides for the CH and Pt electrodes was quantified and normalized using the mean value of the control (unpaired, two-sample *t* test).

### Rat kinematic analysis

Hindlimb kinematic analysis was performed to assess the effects of chronic implantation of electrodes on the locomotor function of the rats. Both the rats with electrodes and the control group without electrodes were kept for 9 weeks following the surgery. Five reflective markers were attached to the iliac crest, hip, knee, ankle, and foot of each rat. A high-speed USB camera (acA1300-200um, Basler, USA) operated at 120 Hz was set perpendicular to the side of a treadmill at a distance of 60 cm to record the two-dimensional positions of the reflective markers while the rats walked on the treadmill. An light-emitting diode lamp (led-7100T, Genaray, USA) was positioned behind the camera to create optimal lighting for recording. The stick diagrams of the hindlimb extracted from the recorded video were used to quantify the knee and ankle translations.

### In vivo electrical stimulation and CMAP recording

The Institutional Animal Care and Use Committee (IACUC) at Texas A&M University reviewed and approved all protocols related to stimulation and implantation in rats (IACUC 2024-0005). Male Sprague-Dawley rats from Envigo (Houston, TX) were used for these experiments. All the tools and electrodes were sterilized. Initially, 5% isoflurane gas was used to anesthetize the rats. Later, the isoflurane concentration was slowly reduced to 2 to 3%. A heating pad was laid under the rats to keep the rats warm at 37°C. After removing hairs on the thigh of the hind leg, an ~2-cm incision was made, and the sciatic nerve was exposed by separating the femoral muscles. The sterilized forceps were used to lift the sciatic nerve to wrap the encapsulated electrodes around the nerve. A biphasic, symmetric square-wave voltage pulse train with a pulse width of 200 μs at 100 Hz and an intertrain duration of 5 s was applied to stimulate the sciatic nerve. We increased the voltage amplitude from 0.1 to 0.5 V and quantified the resultant ankle movement. To record CMAP evoked by electrical nerve stimulation, the device containing three electrodes (working, reference, and ground) was conformably laminated on the muscle surface near the sciatic nerve. The CMAP was recorded following stimulation with increasing amplitudes. After the stimulation and CMAP recording, surgical sutures were used to close the incisions.
