# Supplementary material for: Soft, stretchable conductive hydrogels for high-performance electronic implants
Source: Sci Adv. 2025 Mar 21;11(12):eads4415. doi: 10.1126/sciadv.ads4415 (PMC11927610; doi:10.1126/sciadv.ads4415)
Supplement: Supplementary file 1 — Table S1 Figs. S1 to S18 [file sciadv.ads4415_sm.pdf]

Science Advances
AAAS

Supplementary Materials for

Soft, stretchable conductive hydrogels for high-performance electronic implants

Md Saifur Rahman *et al.*

Corresponding author: Limei Tian, ltian@tamu.edu

Sci. Adv. **11**, eads4415 (2025)
DOI: 10.1126/sciadv.ads4415

This PDF file includes:

Table S1
Figs. S1 to S18

Table S1. Comparison of conductive hydrogels (CH) for electrostimulation.

Note: our CH electrodes yield a higher CSC, CIC, and ankle rotation angle (highlighted in bold) than previously reported hydrogel electrodes.

Additives in conductive hydrogels (reference number)	Electrical conductivity in DI water, S cm^{-1}	Young's Modulus in DI water, Mpa	Stretchability, %	CSC, mC cm^{-2}	CIC, mC cm^{-2} with pulse duration 100 ms	Impedance at 1kHz, ohms	Stimulation parameter: amplitude, duration, frequency	Ankle rotation angle° (% of maximum angle)
DMSO (7)	~ 40	~ 2 (Tensile)	~ 25	60	8	NA	NA	NA
Ionic liquid (8)	~ 47	0.032 (Compression)	20	164	NA	~500	0.1 V, 200 µs, 100 Hz	~ 30%
Laser/Gold nanoparticle (14)	~ 670	57 (Tensile)	20	32	NA	5000	0.5 V, NA, 10 Hz	~ 3°
Laser/ ethylene glycol (16)	101.4	~ 15 (Tensile)	~ 13	49	NA	104	NA	NA
DMSO (17)	~ 11 (PBS)	~ 1 (Tensile, PBS)	~ 400	6	7	~ 80	1 mA, 200 µs, 1 Hz	~ 20°
DMSO (18)	~ 100	0.025 (Tensile)	~ 200	80	NA	~ 28	0.1 V, 25000 µs, 1 Hz	~ 10°
Acetic acid (19)	~10	0.4 (Tensile)	~ 150	48	0.25 (40 ms)	~ 38	0.125 V, 25000 µs, 1 Hz	~ 1°
D-sorbitol (this work)	~ 74	2.6 (Tensile), 0.7 (Compression)	~ 40	**172**	**47**	79	0.1 V, 200 µs, 100 Hz	**51° (100%)**

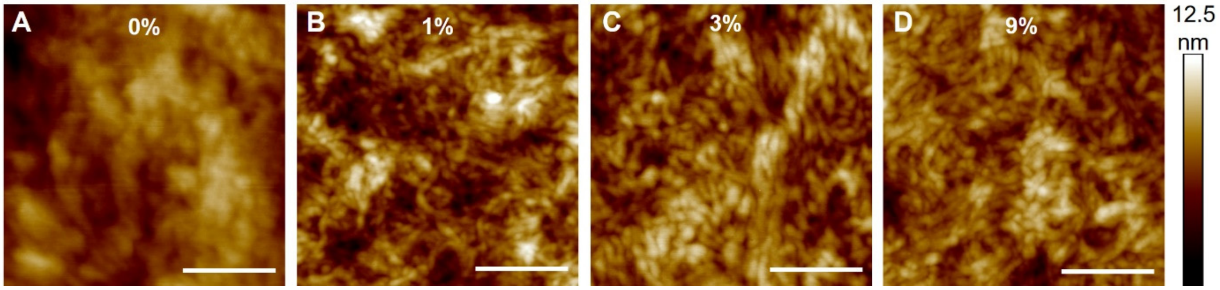

Figure S1. AFM height sensor without D-sorbitol treated and different concentrations of D-sorbitol treated dry-annealed PEDOT:PSS freestanding films. (**A**) 0%, (**B**) 1 w/v%, (**C**) 3 w/v%, and (**D**) 9 w/v%. The measured surface roughness is (**A**) 2.09 nm, (**B**) 1.25 nm, (**C**) 1.57 nm, and (**D**) 1.73 nm. Scale bars 100 nm.

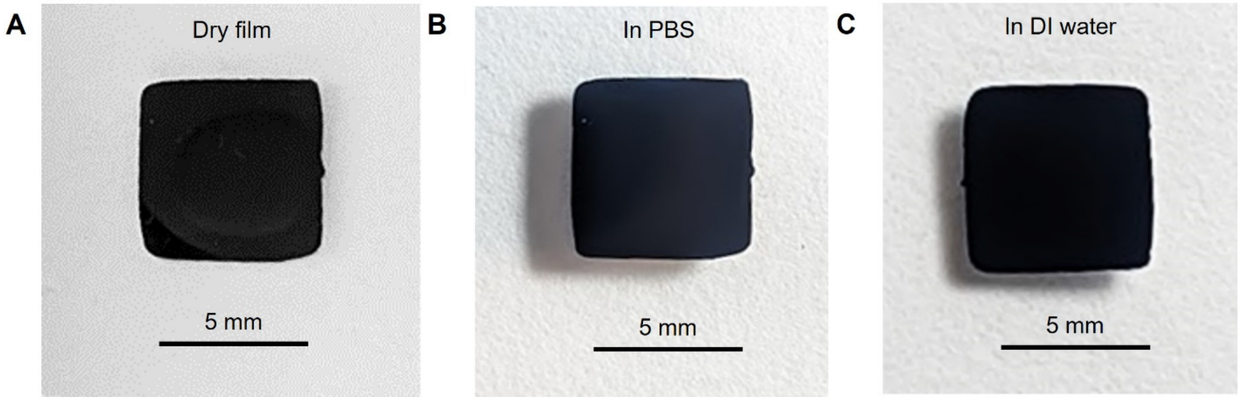

Figure S2. Optical images of (**A**) PEDOT:PSS film, (**B**) hydrogel films immersed in 1X PBS after 3 months, and (**C**) hydrogel films immersed in DI water after 3 months.

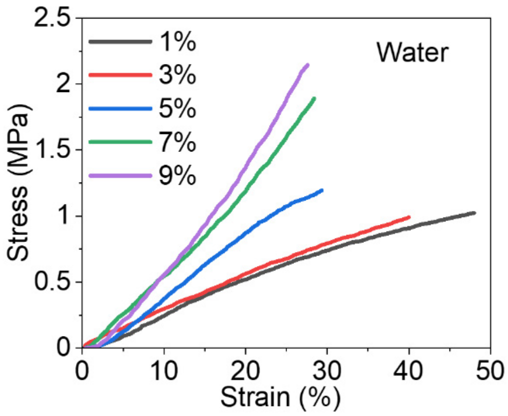

Figure S3. Stress vs. strain curves of freestanding CH in water with varying D-sorbitol concentrations.

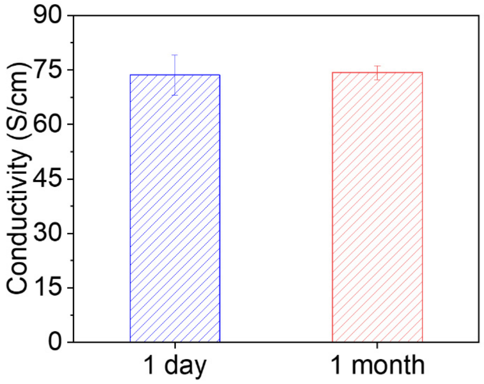

Figure S4. Electrical conductivity of the CH after soaking in water for 1 day and 1 month.

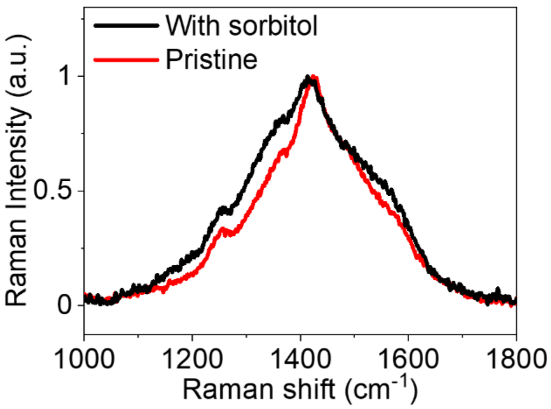

Figure S5. Raman spectroscopy of pristine PEDOT:PSS films and treated with 3 w/v% D-sorbitol.

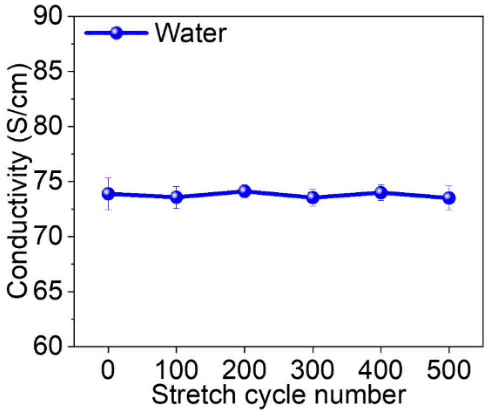

Figure S6. CH electrical conductivity after numerous stretch and release cycles at 30% strain.

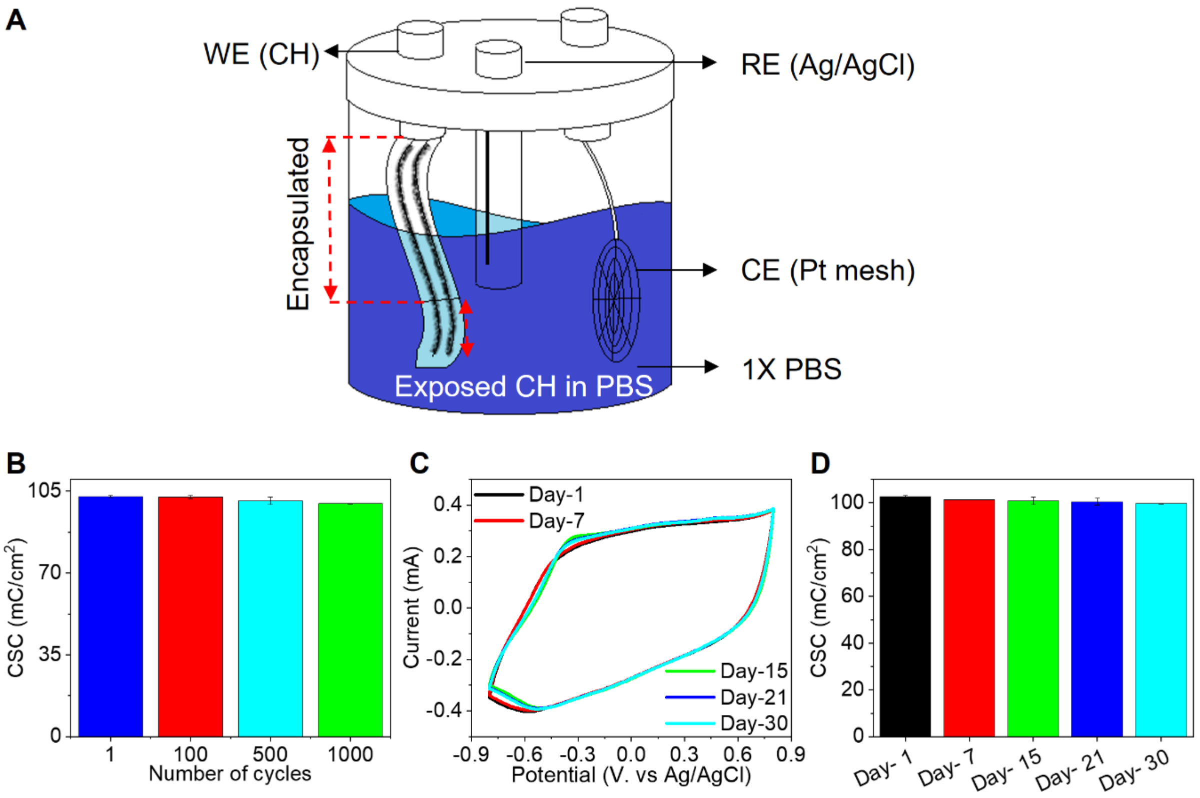

Figure S7. Electrochemical stability of CH before autoclave. (A) Representation of the three electrodes- set up used for electrochemical analysis, exposed GSA only used for calculation. Ag/AgCl and Pt mesh were used as a reference and counter electrode, respectively. **(B)** Electrochemical stability CH upon 1000 CV cycles with scan rate 100 mV s^{-1} (n=3). **(C, D)** Electrochemical stability of CH over time, showing good stability of CH for one month (n=3). All error bars denote the SD.

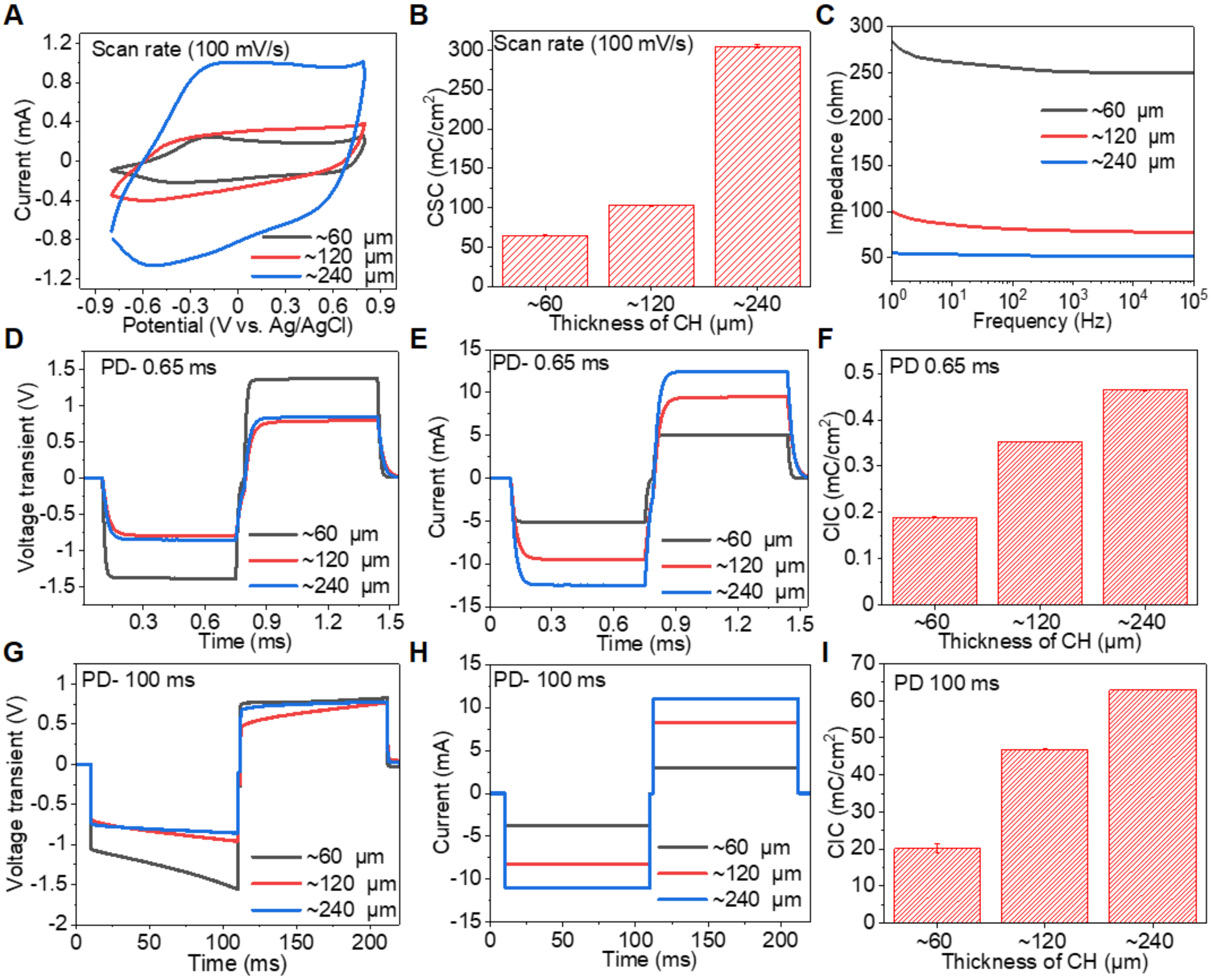

Figure S8. Electrochemical properties of CH electrodes of different thicknesses. (A) CV curves of CH electrodes recorded at a 100 mV/s scan rate and **(B)** corresponding CSC. **(C)** Impedance magnitude of CH electrodes. **(D)** Voltage transient curves, **(E)** corresponding current pulses with a pulse duration of 650 µs, and **(F)** calculated CIC. **(G)** Voltage transient curves, **(H)** corresponding current pulses with a pulse duration of 100 ms, and **(I)** calculated CIC. (n=3).

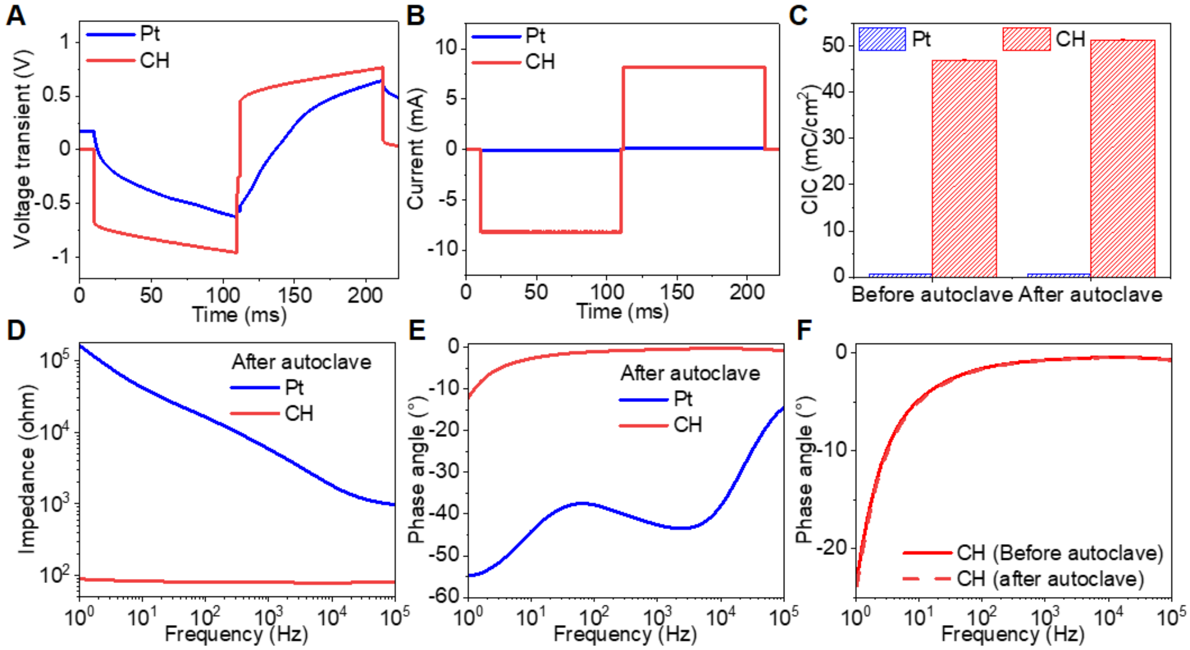

Figure S9. Electrochemical properties of CH. (**A**) Voltage transient curves of the CH electrodes prepared with varying concentrations of D-sorbitol from 1 w/v% to 9 w/v%, (**B**) corresponding current pulses with pulse duration of 100 ms, and **(C)** calculated CIC of CH and Pt electrodes before and after autoclave (n=3). **(D, E)** bode plot and the corresponding phase angle diagrams of CH and Pt electrodes after autoclave. **(F)** phase angle diagrams of CH and Pt electrodes before and after autoclave. All error bars denote the SD.

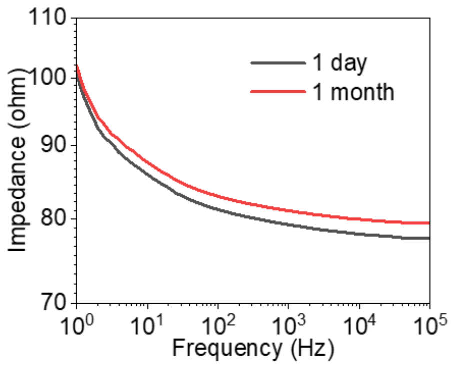

Figure S10. The impedance of hydrogel electrode after soaking in water for 1 day and 1 month.

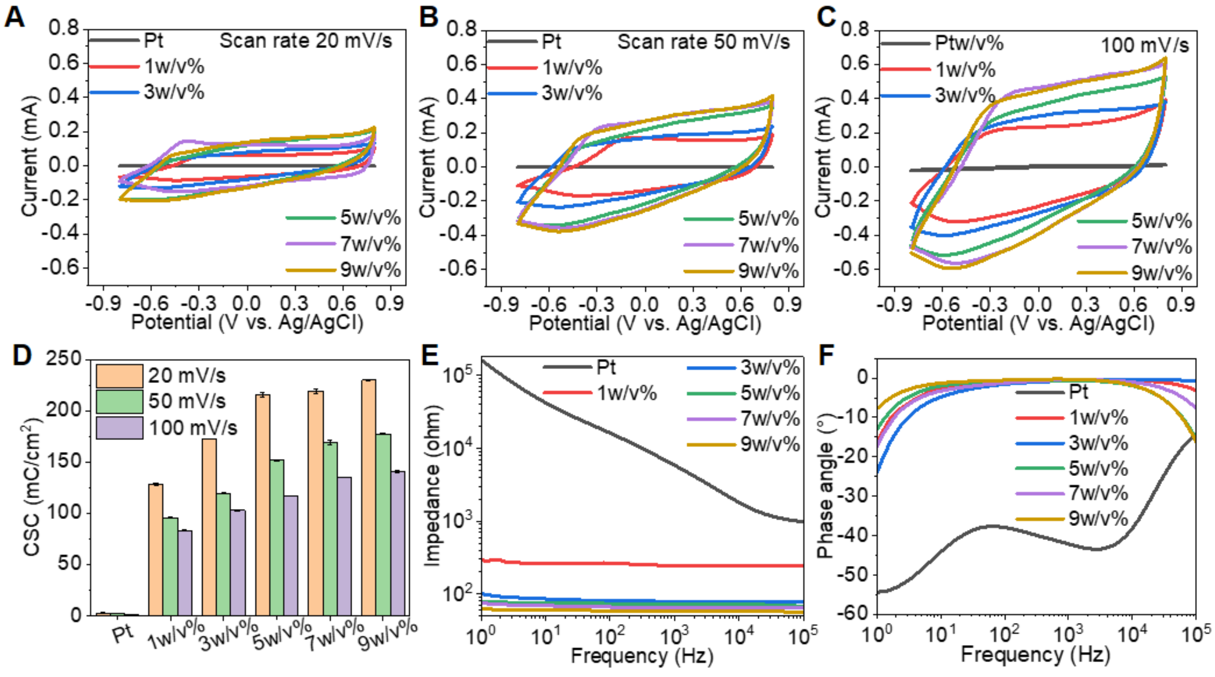

Figure S11. CSC and impedance of the CH electrodes prepared with varying concentrations of D-sorbitol from 1 w/v% to 9 w/v%. (A, B, and C) CV curves of CH electrodes recorded with varying scan rates of 20, 50, and 100 mV/s and **(D)** corresponding CSC. **(E)** Impedance magnitude and **(f)** phase angle plots of the CH electrodes with varying frequency.

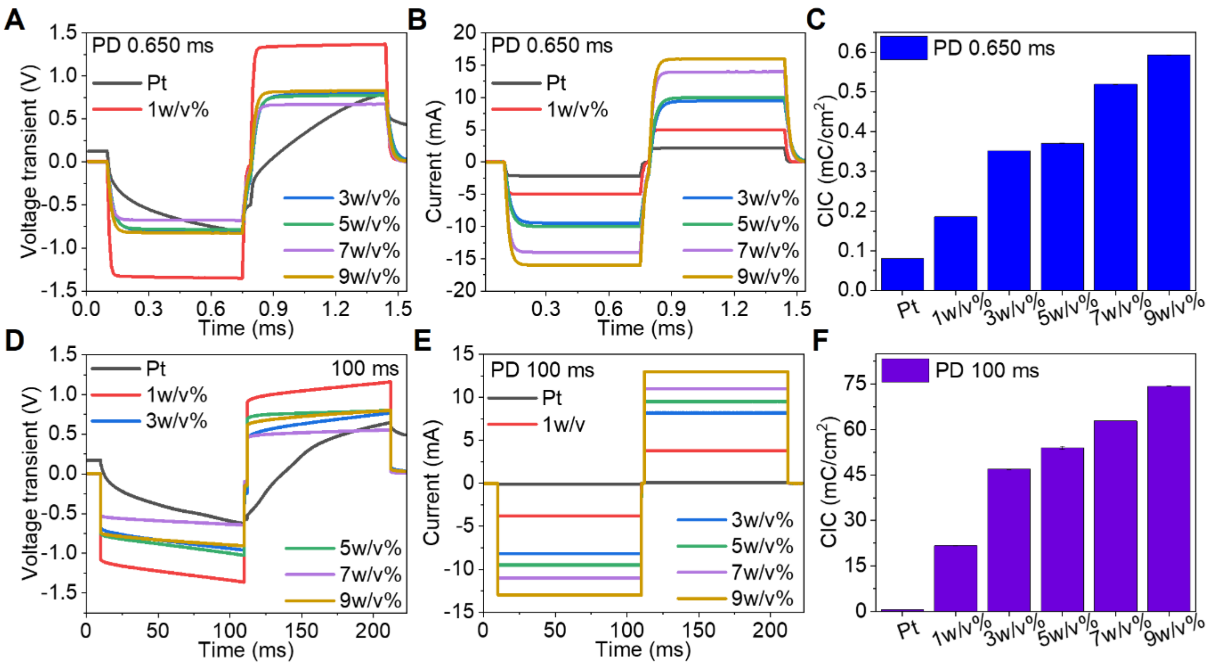

Figure S12. CIC characterization. (A) Voltage transient curves of the CH electrodes prepared with varying concentrations of D-sorbitol from 1 w/v% to 9 w/v%, **(B)** corresponding current pulses with a pulse duration of 0.650 ms, and **(C)** calculated CIC. **(D)** Voltage transient curves of the CH electrodes on PDMS, Au backing, and the freestanding CH electrode **(E)** corresponding current pulses with a pulse duration of 100 ms, and **(F)** calculated CIC.

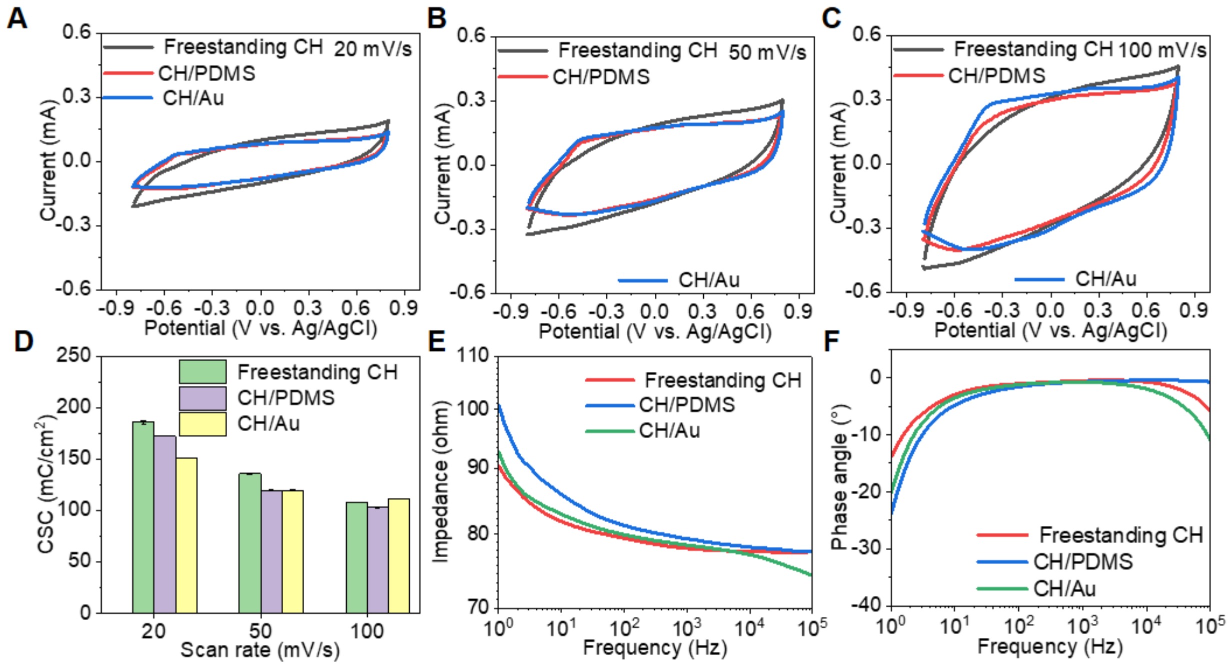

Figure S13. CSC and impedance of the CH electrodes on PDMS, Au backing, and the freestanding CH electrode. (A, B, and C) CV curves of CH electrodes recorded with varying scan rates of 20, 50, and 100 mV/s and **(D)** corresponding CSC. **(E)** Impedance magnitude and **(F)** phase angle plots of the CH electrodes with varying frequency.

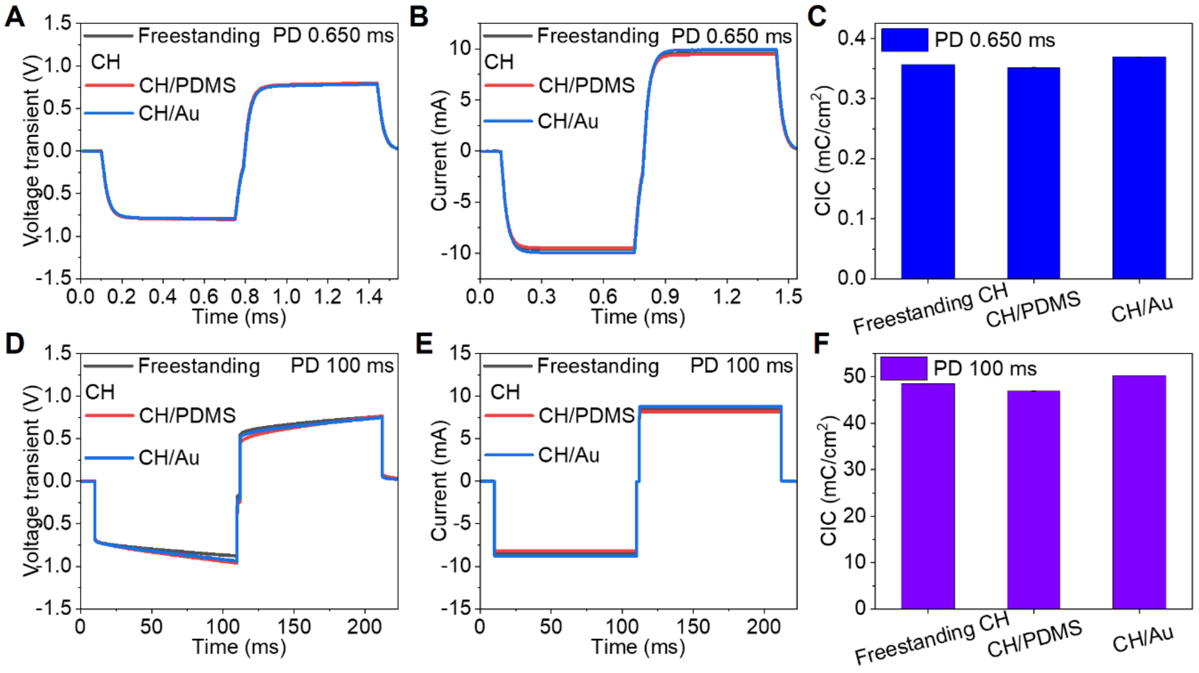

Figure S14. CIC characterization. (A) Voltage transient curves of the CH electrodes on PDMS, Au backing and the freestanding CH electrode, (B) corresponding current pulses with a pulse duration of 0.650 ms, and (C) calculated CIC. (D) Voltage transient curves of the CH electrodes on PDMS, Au backing, and the freestanding CH electrode (E) corresponding current pulses with a pulse duration of 100 ms, and (F) calculated CIC.

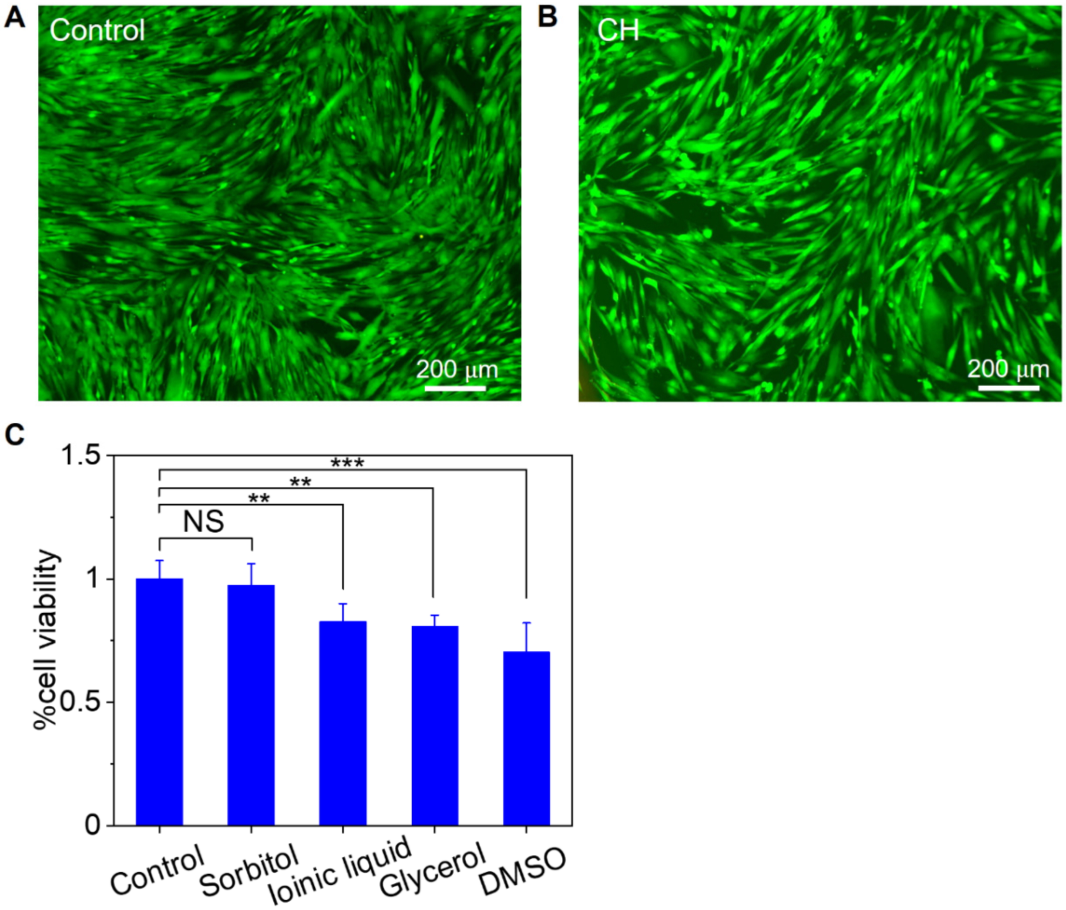

Figure S15. Cytotoxicity characterization. (A, B) Live/dead assay after 72 hrs culture (green fluorescence represents a live cell, and red fluorescence represents a dead cell). (**C**) MTT assay quantified cell viability after exposure to the additive-containing media for 24 hours. The P values for comparison of the cell viability are as follows: control vs. D-sorbitol, $P = 0.61283$, control vs. ionic liquid, $P = 0.00514$, control vs. glycerol, $P = 0.00177$, and control vs. DMSO, $P = 0.00034$ (n=3). ** $P \leq 0.01$, and *** $P \leq 0.001$. NS represents not significant.

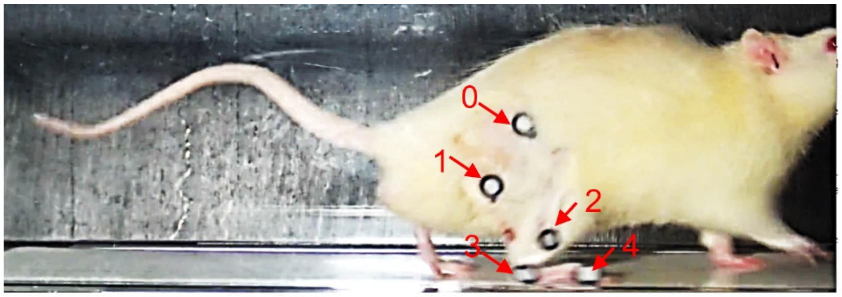

0: Iliac crest 1: Hip 2: Knee 3: Ankle 4: Foot

Figure S16. Optical image of a rat with the attached reflective markers for kinematic analysis after nine weeks of implantation.

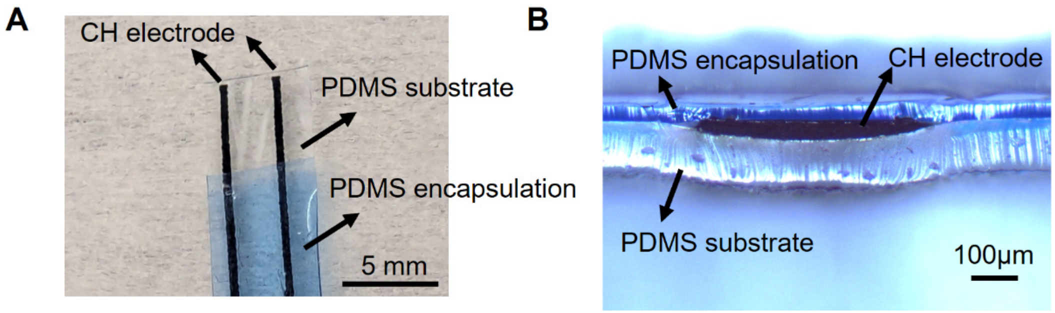

Figure S17. CH electrode for stimulation. (A) Optical image of CH electrode. Colored PDMS is used to show a clear view of the thin encapsulation layer of PDMS. **(B)** Optical microscopic image showing encapsulation of CH between the PDMS layers.

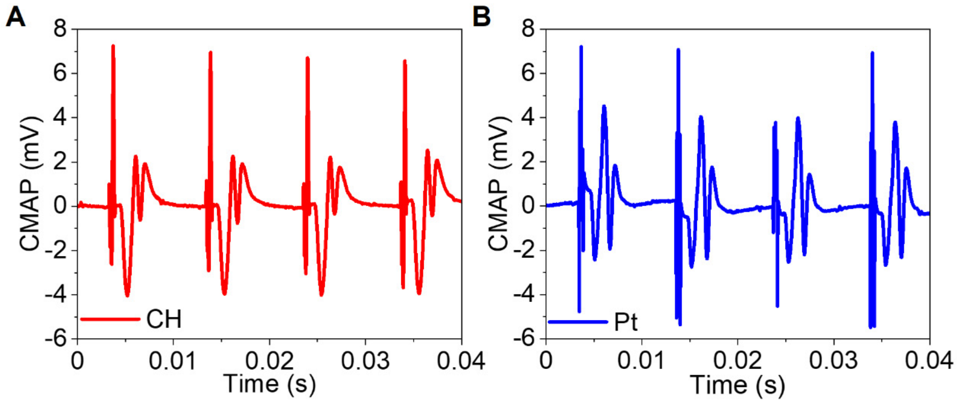

Figure S18. 0.3 V stimulation evoked CMAP recorded using (**A**) CH electrodes and (**B**) Pt electrodes.
